# LRRK2 kinase activity and biology are not uniformly predicted by its autophosphorylation and cellular phosphorylation site status

**DOI:** 10.3389/fnmol.2014.00054

**Published:** 2014-06-24

**Authors:** April Reynolds, Elizabeth A. Doggett, Steve M. Riddle, Connie S. Lebakken, R. Jeremy Nichols

**Affiliations:** ^1^Parkinson’s InstituteSunnyvale, CA, USA; ^2^Thermo Fisher ScientificMadison, WI, USA

**Keywords:** LRRK2, Parkinson’s disease, kinase, GTPase, phosphorylation, kinase inhibitor

## Abstract

Missense mutations in the Leucine-Rich Repeat protein Kinase 2 (LRRK2) gene are the most common genetic predisposition to develop Parkinson’s disease (PD) (Farrer et al., 2005; Skipper et al., 2005; Di Fonzo et al., 2006; Healy et al., 2008; Paisan-Ruiz et al., 2008; Lesage et al., 2010). LRRK2 is a large multi-domain phosphoprotein with a GTPase domain and a serine/threonine protein kinase domain whose activity is implicated in neuronal toxicity; however the precise mechanism is unknown. LRRK2 autophosphorylates on several serine/threonine residues across the enzyme and is found constitutively phosphorylated on Ser910, Ser935, Ser955, and Ser973, which are proposed to be regulated by upstream kinases. Here we investigate the phosphoregulation at these sites by analyzing the effects of disease-associated mutations Arg1441Cys, Arg1441Gly, Ala1442Pro, Tyr1699Cys, Ile2012Thr, Gly2019Ser, and Ile2020Thr. We also studied alanine substitutions of phosphosite serines 910, 935, 955, and 973 and specific LRRK2 inhibition on autophosphorylation of LRRK2 Ser1292, Thr1491, Thr2483 and phosphorylation at the cellular sites. We found that mutants in the Roc-COR domains, including Arg1441Cys, Arg1441His, Ala1442Pro, and Tyr1699Cys, can positively enhance LRRK2 kinase activity, while concomitantly inducing the dephosphorylation of the cellular sites. Mutation of the cellular sites individually did not affect LRRK2 intrinsic kinase activity; however, Ser910/935/955/973Ala mutations trended toward increased kinase activity of LRRK2. Increased cAMP levels did not lead to increased LRRK2 cellular site phosphorylation, 14-3-3 binding or kinase activity. In cells, inhibition of LRRK2 kinase activity leads to dephosphorylation of Ser1292 by Calyculin A and Okadaic acid sensitive phosphatases, while the cellular sites are dephosphorylated by Calyculin A sensitive phosphatases. These findings indicate that comparative analysis of both Ser1292 and Ser910/935/955/973 phosphorylation sites will provide important and distinct measures of LRRK2 kinase and biological activity *in vitro* and *in vivo*.

## Introduction

Parkinson’s disease (PD) is a progressive neurodegenerative disease affecting 1–2% of the population over 65 years of age, with approximately 60,000 newly diagnosed patients per year. It is estimated that the prevalence of PD cases worldwide will double by the year 2030 (Dorsey et al., 2007). The increasing disability caused by the progression of disease burdens the patients, their caregivers, as well as society. Hallmark clinical features of PD include resting tremor, bradykinesia, postural instability and rigidity. PD also exhibits a wide variety of non-motor features such as autonomic dysfunction and dementia. Although the pattern of neuronal loss in PD is well-characterized, the molecular mechanisms of progressive cell death are still being elucidated. The majority of PD patients suffer from idiopathic disease with no clear etiology. However, approximately 5% of patients present with familial PD (Zimprich et al., 2004; Di Fonzo et al., 2005, 2006; Farrer et al., 2005; Healy et al., 2008; Paisan-Ruiz et al., 2008; Simon-Sanchez et al., 2009). Furthermore, exposure to a number of environmental toxicants has been shown to increase the risk of PD. Therefore, understanding the mechanisms of these known causes and risks will likely lead to the development of novel therapeutics, an unmet need.

Insights provided by understanding biochemical and cellular functions of the normal as well as mutated PD genes can provide insights into the pathogenesis of both inherited and idiopathic PD, because mutations in the leucine-rich repeat kinase 2 gene (*LRRK2*) are a common cause of inherited and idiopathic forms of PD. Genome-wide association studies have also identified LRRK2 as a risk factor for sporadic PD (Satake et al., 2009; Simon-Sanchez et al., 2009; Ross et al., 2011; Lill et al., 2012). The informative nature of PD causing mutations on the molecular basis of disease is demonstrated by LRRK2, because clinical phenotypes of PD caused by LRRK2 mutation are largely indistinguishable from idiopathic disease (Ishihara et al., 2006; Ross et al., 2006; Haugarvoll et al., 2008; Haugarvoll and Wszolek, 2009), however ascertainment of larger LRRK2 Gly2019Ser patient populations will certainly define distinct clinical and pathologic features of LRRK2 parkinsonism (Zimprich et al., 2004; Adams et al., 2005; Whaley et al., 2006; Sossi et al., 2010).

LRRK2 encodes a large multi-domain protein with both a GTPase and a kinase domain. The amino terminus [1–1287 aa] is dispensable for kinase activity (Jaleel et al., 2007) but participates in regulation of LRRK2. This region of LRRK2 contains a phosphorylation cluster, an armadillo domain, an ankyrin domain, and a leucine-rich repeat domain. The remaining protein [1335–2527aa] consists of the minimal catalytic fragment, which includes the GTPase domain termed Ras of complex proteins (Roc), followed by the C-terminal-of-Roc domain (COR), which is contiguously connected to the N-terminus of the kinase domain. The kinase domain bears similarity to mixed lineage kinases, which are typically involved in kinase signaling cascades; however no upstream or downstream kinases have been validated yet. The carboxy terminus contains a WD40 domain and is essential for kinase activity. Deletion of the carboxy terminus or substitution of the risk factor mutation Gly2385Arg decreases kinase activity of LRRK2 (Jaleel et al., 2007; Greggio et al., 2008; Jorgensen et al., 2009; Rudenko et al., 2012). Many of the LRRK2 substitutions described to be pathogenic are concentrated in the catalytic tri-domain, including the Asn1437His, Arg1441Cys, Arg1441Gly, Arg1441His, and Tyr1699Cys in the Roc and COR domains, and Gly2019Ser and Ile2020Thr in the kinase domain (Biskup and West, 2009). The Arg1628Pro risk factor mutation falls within the COR domain (Ross et al., 2008; Tan et al., 2008). The most common mutation in inherited and idiopathic PD encodes Gly2019Ser, and is located in subdomain VII of the kinase domain and has consistently been shown to increase kinase activity 2–3 fold (Paisan-Ruiz et al., 2004; Zimprich et al., 2004; West et al., 2005; Jaleel et al., 2007). Other pathogenic substitutions, Arg1441Cys, Arg1441Gly, Arg1441His, Tyr1699Cys, and Ile2020Thr, modestly increase kinase activity and GTPase activity (West et al., 2005, 2007; Greggio and Cookson, 2009; Nichols et al., 2010; Webber et al., 2011).

LRRK2 kinase is itself a phosphoprotein that is regulated by upstream kinases and autophosphorylation. LRRK2 autophosphorylation has been observed on more than 20 threonine and serine residues with a preference for threonine (Kamikawaji et al., 2009; Gloeckner et al., 2010; Li et al., 2010; Pungaliya et al., 2010). Supplemental Table 1 gathers the reported autophosphorylation sites of LRRK2 from several publications. An Ala substitution of Thr1503 decreases GTPase activity and kinase activity (Webber et al., 2011). Autoregulation of Thr1348 or Thr1349 affects GTP binding and kinase activity (Kamikawaji et al., 2013). Autophosphorylation of Ser1292 has recently been observed within cells as an indicator of LRRK2 kinase activity through autophosphorylation (Sheng et al., 2012); however the comparative utility to other autophosphorylation sites has yet to be explored. In the absence of a validated downstream substrate for LRRK2, autophosphorylation is a potential tool to understand LRRK2 kinase activity in experimental or pathological conditions.

A cluster of serines, including Ser910, Ser935, Ser955, and Ser973, found preceding the namesake LRR domain, appears to be constitutively phosphorylated on LRRK2. It has been proposed that these sites are phosphorylated by kinases other than LRRK2 itself (West et al., 2007; Gloeckner et al., 2010; Nichols et al., 2010; Li et al., 2011; Doggett et al., 2012) and are referred to as the cellular phosphorylation sites herein. These sites are dynamically regulated, becoming rapidly dephosphorylated in cells and tissues after inhibition of LRRK2 with small molecule kinase inhibitors. This has been utilized as an indirect measure of LRRK2 activity in cells and tissues by multiple groups. The phosphorylation of LRRK2 is also implicated in mutation induced disease, as PD-associated mutations Asn1437His, Arg1441Cys, Arg1441Gly, Arg1441His, Tyr1699Cys, and Ile2020Thr all show decreased phosphorylation at the cellular phosphorylation sites Ser910/935/955/973. Dephosphorylation of the cellular sites links PD mutations and inhibition with similar molecular outcomes such as relocalization to cytoplasmic accumulations and filamentous skein like structures, loss of 14-3-3 binding and increased binding of PP1α (Greggio et al., 2006; Alegre-Abarrategui et al., 2009; Nichols et al., 2010; Kett et al., 2011; Lobbestael et al., 2013).

LRRK2 activity is regulated by inputs from other domains; these could be intramolecular or intermolecular via domain-domain interactions or oligomerization. The GTPase activity of Roc influences kinase activity of the kinase domain, while the carboxy terminus is also necessary for kinase activity. The PD-associated mutation Gly2385Arg or deletion of only the last seven amino acids in the C-terminus of LRRK2 leads to downregulation of kinase activity. PD-associated mutations of the Roc domain [Arg1441Cys/Arg1441Gly/Arg1441His] or the COR domain [Tyr1699Cys], which showed reduced GTPase activity and/or impaired dimerization, also impact kinase activity of LRRK2. The kinase domain is thought to signal back to the amino terminus via a feedback phosphorylation mechanism of an intermediate kinase(s). Analysis of autophosphorylation mutants can help determine how each domain can regulate activity of LRRK2. Herein, we elucidate differential effects of PD mutations in the Roc-COR, kinase, and carboxy terminus of LRRK2 on the autophosphorylation at Ser1292, Thr1491, and Thr2483 with respect to cellular phosphorylation at Ser910/935/955/973. These outputs were also analyzed in phosphosite mutations of the amino terminus. We found that Ser1292 autophosphorylation is effective at detecting kinase activity in cells and reveals increased kinase activity of a heterozygous Gly2019Ser mutation in patient derived, Epstein-Barr virus (EBV) immortalized lymphoblasts. However, Ser1292 autophosphorylation is inversely regulated with the cellular site phosphorylation readouts in several PD associated mutations. We clearly show that Roc-COR PD mutations increase kinase activity and also decreased phosphorylation of the cellular sites. Alanine substitutions of Ser1292, Thr1491, Thr2483, Ser910/Ser935, Ser955, or Ser973 did not decrease LRRK2 kinase activity, nor did Ser1292, Thr1491, and Thr2483 to Ala substitutions decrease cellular site phosphorylation. We found that increased cyclic adenosine-monophosphate (cAMP) does not lead to increased phosphorylation of Ser1292, Ser910, Ser935, Ser 955, or Ser973 in HEK293 cells. Additionally, we found that Ser1292 is regulated by Okadaic acid and Calyculin A sensitive phosphatases in contrast to the cellular sites which are only subject to regulation by Calyculin A sensitive phosphatases.

## Materials and methods

### Reagents and general procedures

Tissue culture reagents were from Life Technologies or Thermo Scientific. The Flp-in T-REx system was from Invitrogen and stable cell lines were generated as per manufacturer instructions by selection with hygromycin as has been described previously (Nichols et al., 2010; Doggett et al., 2012). Restriction enzyme digests, DNA ligations, and other recombinant DNA procedures were performed using standard protocols with Fermentas enzymes. DNA constructs used for transfection were purified from Escherichia coli DH5a using Qiagen plasmid Maxi kits or Invitrogen Maxi pep kits according to the manufacturer’s protocol. All DNA constructs and DNA transfections were performed using the polyethylenimine method according to Reed et al. (2006).

All DNA constructs used for transfections were provided by Dr. Dario Alessi (MRC-PPU, Dundee University, Dundee Scotland), except the autophosphorylation plasmids (FLAG-Thr1491, FLAG-Th2483, FLAG-S1292). These plasmids and the alanine substitution mutants of the autophosphorylation mutants were sub-cloned from the corresponding pCMV5-FLAG constructs. All mutagenesis experiments were carried out using the GeneArt site-directed mutagenesis kit (Life Technologies). All DNA constructs were verified by DNA sequencing, performed by Sequetech, Mountain View, CA. Full length amino terminal FLAG tagged LRRK2 was expressed by expressed by BacMam infection of HEK293 cells and purified by immunoaffinity chromatography with anti-FLAG M2. Inhibitor GNE1023 was described in Sheng et al. (2012) and synthesized at Genentech, LRRK2-IN1 was purchased from Tocris and Forskolin and IBMX were from Sigma.

### Buffers

Lysis Buffer contained 50 mM Tris/HCl, pH 7.4, 1 mM EGTA, 1 mM EDTA, 1 mM sodium orthovanadate, 10 mM sodium β-glycerophosphate, 50 mM NaF, 5 mM sodium pyrophosphate, 0.27M sucrose, 1 mM Benzamidine, and 1 mM phenylmethanesulphonylfluoride (PMSF) and was supplemented with 1% Triton X-100. Buffer A contained 50 mM Tris/HCl, pH 7.4, 50 mM NaCl, 0.1 mM EGTA, and 0.27M sucrose. Kinase buffer was 50 mM Tris 7.4, 0.1 mM EGTA.

### Cell culture, treatments and cell lysis

HEK-293 cells were cultured in Dulbecco’s Modified Eagle’s medium supplemented with 10% FBS, 2 mM glutamine and 1× antimycotic/antibiotic solution. HEK-293 T-REx cell lines were cultured in DMEM supplemented with 10% FBS and 2 mM glutamine, 1× antimycotic/antibiotic and 15 μg/ml Blasticidin and 100 μg/ml hygromycin. Cell transfections were performed by the polyethylenimine method (Reed et al., 2006). T-REx cultures were induced to express the indicated protein by inclusion of 1 μg/ml doxycycline in the culture medium for 24 h. Human lung alveolar epithelial A549 cells were cultured in Ham’s F-12 (Kaighn’s) with 10% FBS, 1× antimycotic/antibiotic. After the indicated culture conditions, cells were washed once with PBS and lysed *in situ* with 1.0 ml of lysis buffer per 15 cm dish on ice, then centrifuged at 15,000 × g at 4°C for 15 min. HEK-293 cells transfected with LRRK2 WT and mutant plasmids were lysed 48 h after transfection. Lymphoblastoid cell lines generated by EBV transformation of B lymphocytes were obtained from Coriell Institute for Medical Research. Cell line ND00075 (+/Gly2019Ser) is derived from a donor heterozygous for a G > A transition in exon 42 of LRRK2. Cell line ND03335 is an asymptomatic donor. Human lymphoblastoid cells were maintained in RPMI 1640 with 10% FBS, 2 mM glutamine, 1× antimycotic/antibiotic and were maintained at cell density of 0.3 × 10^6^–2 × 10^6^ cells per ml. Protein concentrations were determined using the Bradford method with BSA as the standard.

### Kinase assays

Kinase assays were set up in a total volume of 50 μl with recombinant LRRK2 or immunoprecipitated LRRK2 as a source of kinase in 50 mM Tris/HCI, pH 7.5, 0.1 mM EGTA, 10 mM MgCl2, and 0.1 mM (γ−^32^P) ATP (500–600 c.p.m/pmol) in the presence of 200 μM LRRKtide peptide substrate. Reactions were incubated at 30°C for the indicated times. Reactions were terminated by addition of LDS protein loading buffer or applying 40 μl of the reaction mixture on to P81 phosphocellulose paper and immersion in 50 mM phosphoric acid. After extensive washing, reaction products were quantified by Cerenkov counting. For autophosphorylation assays, 140 nm of FLAG-LRRK2 was incubated at 30°C for the indicated times in the presence of 10 mM MgCl_2_ and 0.1 mM ATP and stopped by the addition of an equal volume of ice-cold 100 mM EDTA. Reaction products were spotted on nitrocellulose and immunoblotted with FLAG and autophosphorylation site antibodies.

### LRRK2 immunoprecipitation assays

Cell lysates were prepared in lysis buffer (1.0 ml per 15 cm dish) and subjected to immunoprecipitation with anti-FLAG M2 agarose or GFP-Trap A beads (Chromotek) at for 1 h. Beads were washed twice with Lysis Buffer supplemented with 300 mM NaCl, then twice with Buffer A. Immune complexes were either used in kinase assays or incubated at 70°C for 10 min, passed through a Spin-X column (Corning) to separate the eluate from the beads, then boiled in LDS sample buffer. LRRK2 transfected HEK293 cell lysates were subjected to immunoprecipitation as with GFP-Trap beads. For endogenous immunoprecipitation assays, LRRK2 was immunoprecipitated using Anti-LRRK2 (UDD3, Abcam) non-covalently conjugated to protein-A sepharose (1 μg antibody: 1 μl bead) and analyzed by immunoblotting.

### Statistical analysis

For quantification of phosphorylation levels, LRRK2 protein levels were normalized for expression and to the control experimental condition. Statistical analysis was done using GraphPad Prism 6. One-sample *t-*tests using the hypothetical value of 1 for comparison and standard One-Way ANOVA tests were performed with either the Dunnett correction for comparison with a single mean or the Tukey–Kramer correction if every mean is compared individually. *P*-values are designated as ^*^p ≤ 0.05.

## Results

### Differential phospho-regulation of LRRK2 in PD associated mutants

#### In vitro characterization of LRRK2 autophosphorylation in wild type and PD-associated LRRK2 mutants

**Figure 1:**
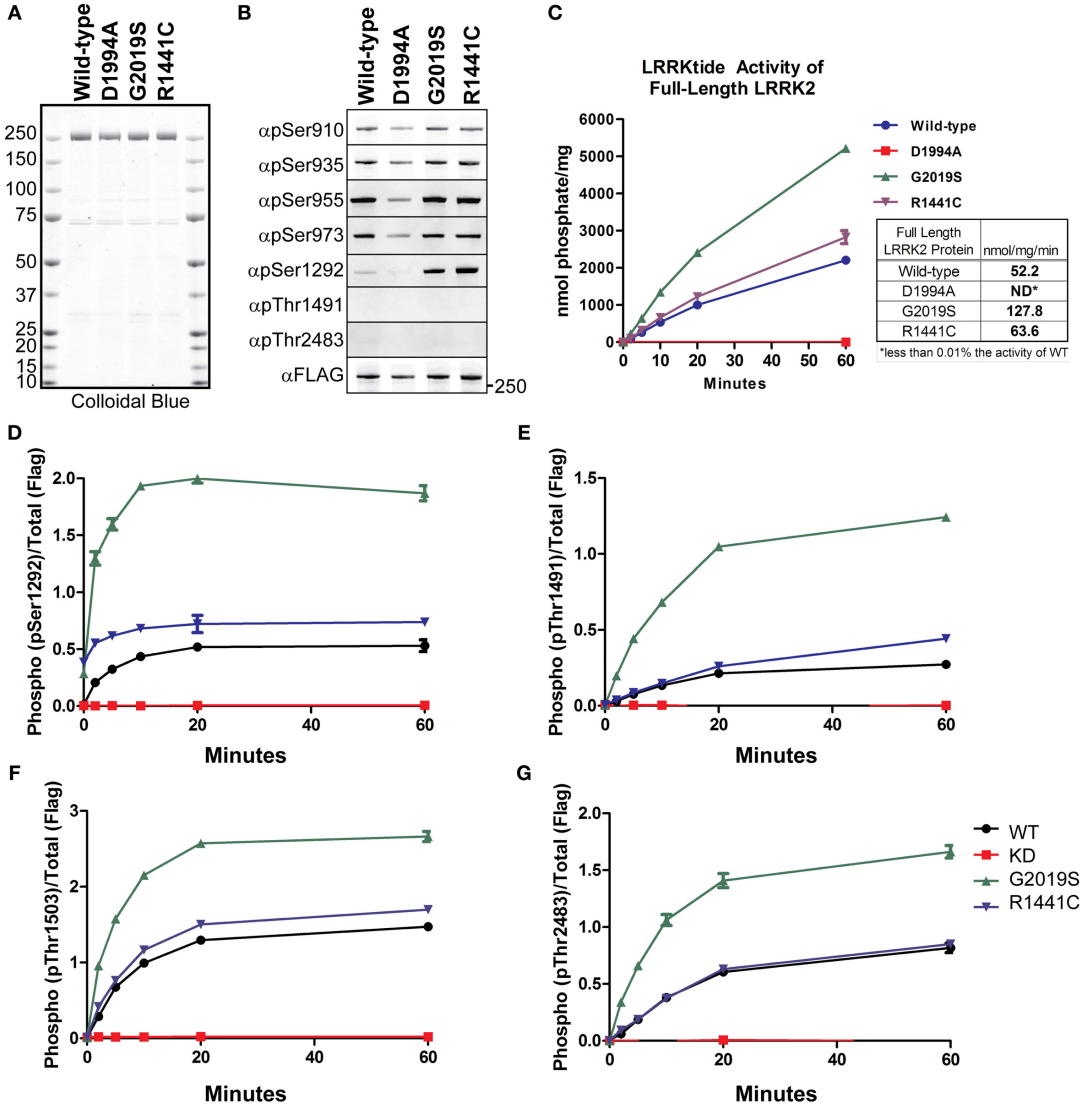
**Detection of autophosphorylation and cellular phosphorylation sites on recombinant LRRK2**. The indicated full-length recombinant LRRK2 proteins were resolved by SDS-PAGE and stained with either **(A)** Colloidal Blue to quantify relative protein levels or **(B)** the indicated phospho-specific antibodies. **(C)** 14 nM of recombinant wild type LRRK2 (WT) or mutant LRRK2 [Asp1994Ala, Gly2019Ser, Arg1441Cys] *in vitro* kinase activity was measured against LRRKtide. Incorporated ^32^P was assessed by Cerenkov counting and results are representative of three independent experiments. Error bars represent mean ± *SD*. **(D–G)** 140 nM recombinant LRRK2 was incubated with Mg^2+^/ATP over a timecourse of 60 min where reaction aliquots were stopped by 1:1 (vol:vol) dilution in 200 mM EDTA and analyzed by dot-blot immunoblot with rabbit monoclonal anti-phospho-Ser1292 (αpS1292), anti-phospho-Thr2483 (αpThr2483), anti-phospho-Thr1491 (αpThr1491) and rabbit polyclonal anti-phospho-Thr1503 antibodies. Results are representative of three independent experiments performed in duplicate and blotted in duplicate. Error bars represent mean ± *SD*.

LRRK2 phosphorylation is a heavily studied aspect of the enzyme’s regulation. LRRK2 phosphosites have been identified from *in vitro* autophosphorylation kinase assays and from enzyme isolated from cells. LRRK2 contains sites of autophosphorylation and also sites that are modified by upstream kinases. We first wished to test the *in vitro* utility of the autophosphorylation site antibodies as indicators of LRRK2 kinase activity in an isolated system, to which we could compare the effects we observe in cellular studies. To detect LRRK2 phosphorylation, we utilized a series of rabbit monoclonal antibodies (anti-pSer910, anti-pSer935, anti-pSer955, anti-pSer973, anti-pThr1491, anti-pThr2483), generated by the Michael J. Fox Foundation and characterized by their Antibodies Working Group, and anti-pSer1292 (generously provided by Genentech), and a rabbit polyclonal anti-pThr1503 [kind gift of Dr. Andrew West, UAB (Webber et al., 2011)]. We verified specificity for the phosphorylation sites with Ser/Thr to alanine mutants, Supplemental Figure 1 and observed no *ab initio* phosphorylation of Thr1491 or Thr2483. We also observed that Ala substitutions at these sites did not negatively impact autophosphorylation at any of the other sites. We next employed full length recombinant LRRK2 [wild-type, Gly2019Ser, Arg1441Cys, and Asp1994Ala] (LifeTechnologies), which are approximately 80% pure as indicated by colloidal blue staining, Figure 1A. Immunoblot analysis of these preparations with anti phospho-Ser910, 935, 955, and 973 antibodies, revealed that phosphorylation of the cellular sites is maintained throughout the purification process and is similar between LRRK2 wild-type and Gly2019Ser. We observed no decrement in the cellular phosphorylation sites of the Arg1441Cys mutation, which has been previously described for Arg1441Gly, Arg1441His substitutions at this residue (Nichols et al., 2010; Doggett et al., 2012). Interestingly, only phosphorylation of the Ser1292 autophosphorylation site was detected in these recombinant protein preparations, as we could not detect *ab initio* phosphorylation of Thr1491 or Thr2483. The PD causing mutations Gly2019Ser and Arg1441Cys exhibited enhanced Ser1292 phosphorylation compared to wild-type, and kinase inactive LRRK2 is not modified at Ser1292 whereas the cellular sites are, Figure 1B.

We characterized the LRRKtide kinase activity of the full LRRK2 length proteins in order to correlate autophosphorylation activity with substrate phosphorylation, Figure 1C. We confirmed that Gly2019Ser mutation exhibited approximately a 2.5 fold increase in specific activity (determined from the linear time points of the reaction) and also observed that the Arg1441Cys mutation increases kinase activity against a peptide substrate approximately 20% over wild-type LRRK2 (Jaleel et al., 2007; Webber et al., 2011), but not at levels equal to Gly2019Ser.

We next compared LRRK2 autophosphorylation at Ser1292 in the amino terminal domain, at Thr1491 and Thr1503 in the central (Roc-COR) region and Thr2483 within the carboxy terminus. We performed *in vitro* autophosphorylation assays with full length LRRK2 in the absence of a substrate and analyzed the ability of the LRRK2 protein to phosphorylate itself over a time course of 60 min using immunoblot of the autophosphorylation sites as a readout. The reaction products were analyzed with anti-pSer1292, pThr1491, pThr1503, and pThr2483 antibodies and quantitated with Odyssey Software (LiCor).

The autophosphorylation sites on LRRK2 were fully modified and reached maximal detection by 20 min, showing similar *in vitro* kinetics of LRRKtide phosphorylation, Figures 1C–G. All antibodies detected a twofold increase in autophosphorylation in the Gly2019Ser mutant and the autophosphorylation activity of the Arg1441Cys mutant is increased relative to wild-type for each antibody tested. Since Ser1292 is phosphorylated at the beginning of the reaction, Gly2019Ser and Arg1441Cys time zero phosphorylation levels are not zero. In contrast to the autophosphorylation sites, the cellular LRRK2 signals did not increase throughout the kinase reaction (data not shown), suggesting that these sites have reached their limit of phosphorylation and further suggesting these are not autophosphorylation sites. Autophosphorylation of LRRK2 and LRRKtide phosphorylation are comparable indicators of LRRK2 kinase activity.

#### Reciprocal regulation of the cellular sites and LRRK2 autophosphorylation

Phosphorylation of the LRRK2 cellular sites is disrupted in the PD mutations Asn1437His, Arg1441Gly, Arg1441His, Tyr1699Cys, and Ile2020Thr (Nichols et al., 2010; Li et al., 2011; Doggett et al., 2012; Lobbestael et al., 2013), while at the same time exhibiting small or enhanced levels of kinase activity (Sheng et al., 2012). To characterize and further understand this dichotomy, we sought to directly compare phosphorylation of Serines910, 935, 955, and 973 with the three autophosphorylation sites Ser1292, Thr1491, and Thr2483 on LRRK2.

**Figure 2:**
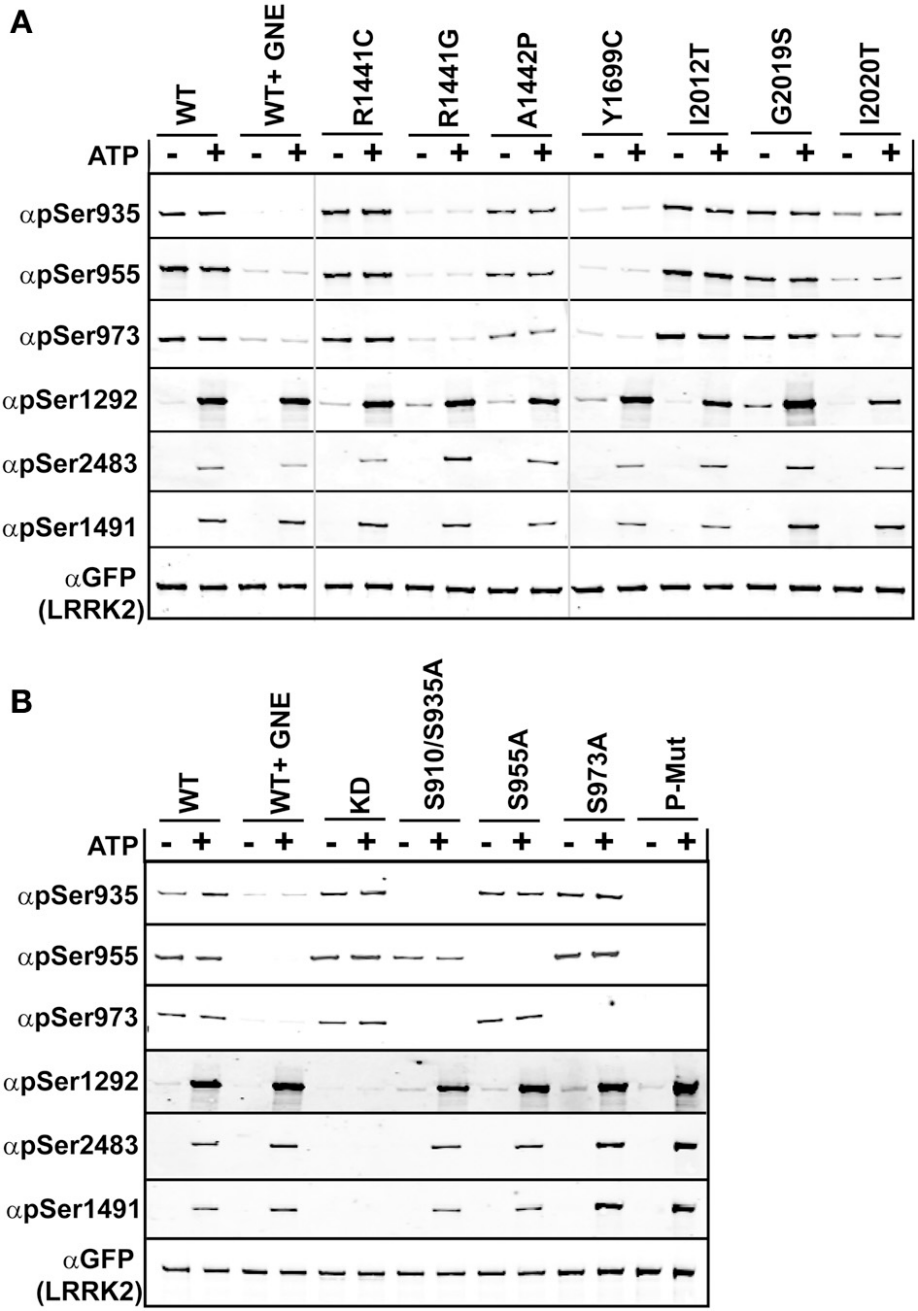
**Comparison of autophosphorylation and cellular phosphorylation sites in PD-associated mutants**. Stable-inducible HEK 293 T-REx cell lines harboring the indicated forms of LRRK2 were incubated for 48 h with 1 μg/ml doxycycline to induce expression. The GFP-LRRK2 WT line was treated with 1 μM GNE1023 for 90 min, before immunoprecipitation-kinase assay using GFP beads, in the presence (+) or absence (−) of ATP. LiCor immunoblot analysis of **(A)** WT and PD associated mutants of LRRK2 and **(B)** WT and cellular phosphorylation mutants. Reaction products were probed with rabbit monoclonal anti-phospho-Ser935 (αpS935), anti-phospho-Ser955 (αpS955), anti-phospho-Ser973 (αp973), anti-phospho-Ser1292 (αpS1292), anti-phospho-Thr2483 (αpThr2483), anti-phospho-Thr1491 (αpThr1491). Blots were probed with anti-GFP (αGFP) for total protein control. Phosphorylation levels are quantitated in Supplemental Figure 2.

We compared the basal phosphorylation in cells to the *in vitro* autophosphorylation of PD mutated LRRK2 using anti-GFP immunoprecipitates from HEK293 T-REx cells expressing GFP-LRRK2 WT, Arg1441Cys, Arg1441Gly, Ala1442Pro, Tyr1699Cys, Ile2012Thr, Gly2019Ser, and Ile2020Thr in a tetracycline inducible manner, Figure 2A. Ala1442Pro is an uncommon mutation (Huang et al., 2007) and has been shown to illicit similar lack of phosphorylation of LRRK2 at the cellular sites and form cytoplasmic accumulations (Greene et al., 2014). Tyr1699Cys is located in the COR domain and also is dephosphorylated at the cellular sites and forms cytoplasmic inclusions, and may impact Roc GTPase activity via decreasing self-association or directly on GTPase activity (Daniels et al., 2011). This mutant will allow us to directly assess the *in cis* influence of Roc-COR domain mutations on the intrinsic kinase activity of LRRK2. The various LRRK2 PD associated mutations were incubated in the presence or absence of Mg^2+^/ATP, where reactions performed in the absence of ATP are representative of the basal state of modification in cells. Reaction products were analyzed by immunoblot with cellular site antibodies and antibodies against Thr1491, Thr2483, and Ser1292; data are presented as quantified LiCor Odyssey values of phospho-signal/total protein set to WT in Supplemental Figure 2. The basal phosphorylation state in the PD-associated mutants showed that Ser1292 and cellular sites (Ser935, 955, and 973) are not uniformly regulated. For mutants Arg1441Cys, Arg1441Gly, Ala1442Pro, and Tyr1699Cys we found that LRRK2 is dephosphorylated at the cellular sites, but is hyperphosphorylated at Ser1292. But like Arg1441Cys behaved *in vitro*, after incubation with ATP, Ser1292 phosphorylation was comparable with WT LRRK2, leaving Gly2019Ser and Y1699C as the only mutants that can incorporate significantly more phosphate at this site from all the mutants tested here. Threonines 1491 and 2483 were evaluated in the presence of ATP and we found that Thr1491 revealed significant increase in modification from the Gly2019Ser mutation, while Thr2483 revealed significant increased kinase activity from Arg1441Gly, and Gly2019Ser. Both Ile2020Thr and Ile2012Thr mutations reduced the basal level of Ser1292 and autophosphorylation activity similar to activity determinations using LRRKtide and Nictide (Jaleel et al., 2007; Nichols et al., 2009). The Ala1442Pro mutant revealed reduced levels of phosphorylation at Thr1491 and Ser1292.

#### Evidence that autophosphorylation and cellular phosphorylation sites are independently regulated, but dependent on LRRK2 kinase activity

Several LRRK2 PD mutations that have exhibited increased Ser1292 phosphorylation (Arg1441Cys, Arg1441Gly, Tyr1699Cys) were dephosphorylated at the cellular sites. Arg1441Gly, Arg1441Cys, and Tyr1699Cys mutants have been shown to form aggregates, which are also seen in the Ser910/935Ala mutant, due to the loss of phospho-dependent interaction with 14-3-3 proteins. We wanted to determine if the Roc-COR mutations effect on kinase activity could be correlated with the loss of cellular phosphorylation. Previous data and Figure 2A indicate that Roc-COR mutations act across the enzyme intramolecularly to the kinase domain to activate the kinase, but also intermolecularly inducing dephosphorylation of the amino terminal cellular sites. We hypothesized that the increase in 1292 phosphorylation in these mutants in cells could be due to intramolecular effects of the amino terminus on the kinase domain. An alternate explanation is there could be an increase in the local concentration or compaction of dephosphorylated LRRK2 in cytoplasmic inclusions that caused more autophosphorylation or less dephosphorylation. We tested these hypotheses on isolated GFP-LRRK2 from HEK293 T-REx cells with stable and inducible expression of LRRK2 cellular phosphosite to alanine mutations [Ser910/935Ala, Ser955Ala, Ser973Ala, and a combination mutant Ser910/935/955/973Ala], Figure 2B, which mimic dephosphorylation of the cellular sites by inhibition or mutation and analyzed LRRK2 phosphorylation after incubation with and without Mg^2+^/ATP in an *in vitro* kinase assay.

As with the recombinant LRRK2 preparations, we found that autophosphorylation of Thr1491 and Thr2483 was only detected after incubation of LRRK2 with Mg^2+^/ATP *in vitro*, in immune-complex kinase assays, Figure 2B and quantitated in Supplemental Figure 2. Mutation of cellular phosphorylation sites [Ser910/935Ala, Ser955Ala, and Ser973Ala] individually had no significant effect on the autophosphorylation of Thr1491 and Thr2483 in the presence of ATP, nor on Ser1292 in cells or *in vitro* in the presence of ATP, Figure 2B. However, in the combination mutant, all autophosphorylation sites trended toward increased kinase activity, (pSer1292 *p* = 0.066; pThr1491 *p* = 0.097; pThr2483 *p* = 0.055). Supplemental Figure 1 also indicates that reciprocal mutation of the autophosphorylation sites does not negatively affect phosphorylation at the cellular sites.

### Analysis of serines 910/935/955/973 and ser1292 dephosphorylation

#### Serines 910/935/955/973 and ser1292 are dephosphorylated after inhibition of LRRK2

In cells, phosphorylation of the cellular sites and autophosphorylation of Ser1292 is dependent on kinase activity of LRRK2. Inhibition of WT LRRK2 with a LRRK2 inhibitor induces the rapid dephosphorylation at the cellular phosphorylation sites (Ser910, 935, 955, 973) and the 1292 site (–ATP lane), Figure 2. To compare the sensitivity of dephosphorylation on serine 1292 vs. the various cellular sites, which can be used to indicate activity of LRRK2, we subjected cells expressing LRRK2 [Gly2019Ser] to a dose response of two structurally distinct LRRK2 inhibitors, LRRK2-IN1 (Deng et al., 2011) and GNE1023 (Estrada et al., 2012; Sheng et al., 2012) for 90 min. Cell lysates were analyzed by immunoblot with antibodies against Ser910, 935, 955, 973 in order to compare the phosphorylation status at each site, LiCor quantitation of immunoblots in Supplemental Figure 3. Both inhibitors induced a dose-dependent dephosphorylation of LRRK2 at the cellular sites and 1292 site, Supplementary Table 1. We find that the shift in biochemical IC_50_ to cellular IC_50_ was on the same order of magnitude for Ser1292 compared to the cellular Ser935, Ser955, and Ser973 sites. We also found that LRRK2 Ala2016Thr mutation blocks the induced dephosphorylation at both types of sites showing that direct inhibition of LRRK2 is required for both dephosphorylation of the autophosphorylation site Ser1292 and the cellular phosphorylation sites, Supplemental Figure 3F. These data indicate that the four cellular phosphorylation sites are effective, though indirect measures of LRRK2 inhibition compared to the direct detection of *in vivo* LRRK2 kinase activity with pSer1292.

#### Comparison of ser1292 and cellular site dephosphorylation in parkinson’s disease patient-derived lymphoblastoid cells

**Figure 3:**
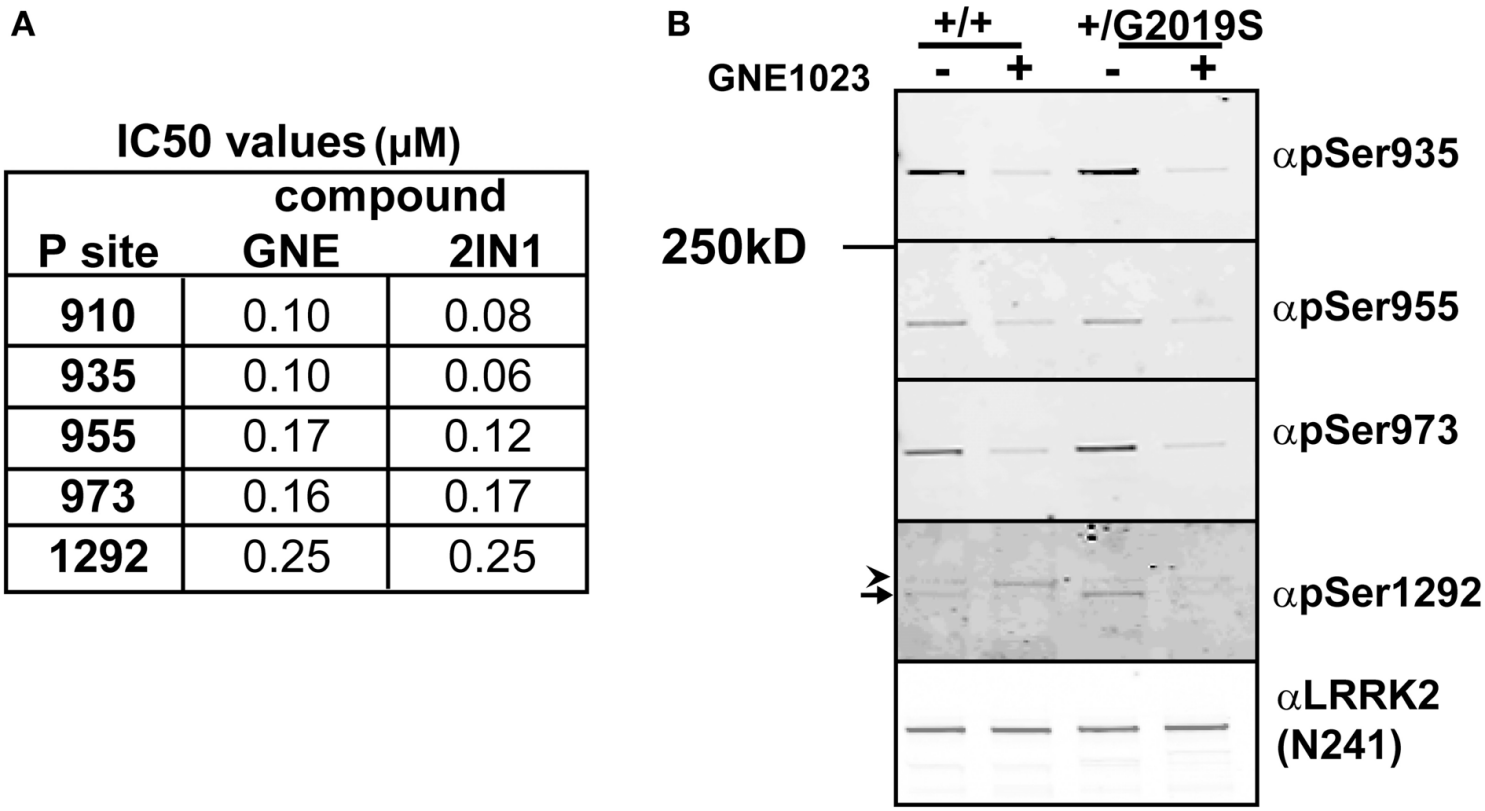
**Comparative analysis of inhibitor induced dephosphorylation for Ser1292 and cellular phosphorylation sites after kinase inhibition. (A)** HEK 293 T-REx cells stably expressing GFP-LRRK2-Gly2019Ser were treated with DMSO or increasing concentrations of GNE1023 or LRRK2-IN1 for 90 min. Cell lysates were co-immunoblotted for GFP and anti-phospho-Ser935 (αpS935), anti-phospho-Ser955 (αpS955), anti-phospho-Ser973 (αp973), anti-phospho-Ser1292 (αpS1292). IC_50_ values were calculated using non-linear regression analysis using GraphPad Prism 6.0. **(B)** Detection of endogenous pSer1292 in human lymphocytes. Cultures of control donor and +/Gly2019Ser donor lymphoblasts were treated with GNE1023 for 90 min. Endogenous LRRK2 was immunoprecipitated and subjected to immunoblot analysis with the indicated phospho-antibodies. Blots were co-probed with N241 monoclonal antibody to detect total endogenous protein. Arrowhead indicates non-specific band and arrow indicates LRRK2.

Since many mutations of LRRK2 increase the kinase activity of the enzyme, LRRK2 inhibitors could serve as a potential preventative or disease modifying drug. In the absence of reliable measures of drug-target engagement such as LRRK2 inhibitor PET ligands, peripheral markers of LRRK2 inhibition could serve as surrogate markers of LRRK2 inhibition in patients. LRRK2 is highly expressed in immune cells and we sought to compare the effectiveness of cellular site and autophosphorylation site antibodies as indicators of LRRK2 activity in EBV transformed lymphoblasts from a control and a PD affected patient heterozygous for Gly2019Ser missense mutation. The cells were treated for 90 min with 2 μM GNE1023 before endogenous LRRK2 was immunoprecipitated and equal amounts of protein were immunoblotted for pSer1292 and Ser935, Ser955, and Ser973 cellular phosphosite antibodies, Figure 3B. Similar levels of LRRK2 were found in the wild type and Gly2019Ser cell line. We were able to detect Ser1292 in the WT lymphoblasts (arrow in Figure 3B), with an increase in pSer1292 observed from the heterozygous Gly2019Ser cells, Figure 3. The increase in Ser1292 signal therefore reveals upregulated LRRK2 activity in Gly2019Ser lymphoblasts. Furthermore, the cells responded to treatment with GNE1023 in culture, showing dephosphorylation at both autophosphorylation and cellular sites, further validating the potential of these sites as potential pharmacodynamic markers of LRRK2 inhibition in patients.

#### cAMP does not increase LRRK2 phosphorylation in cells of kidney or lung origin

**Figure 4:**
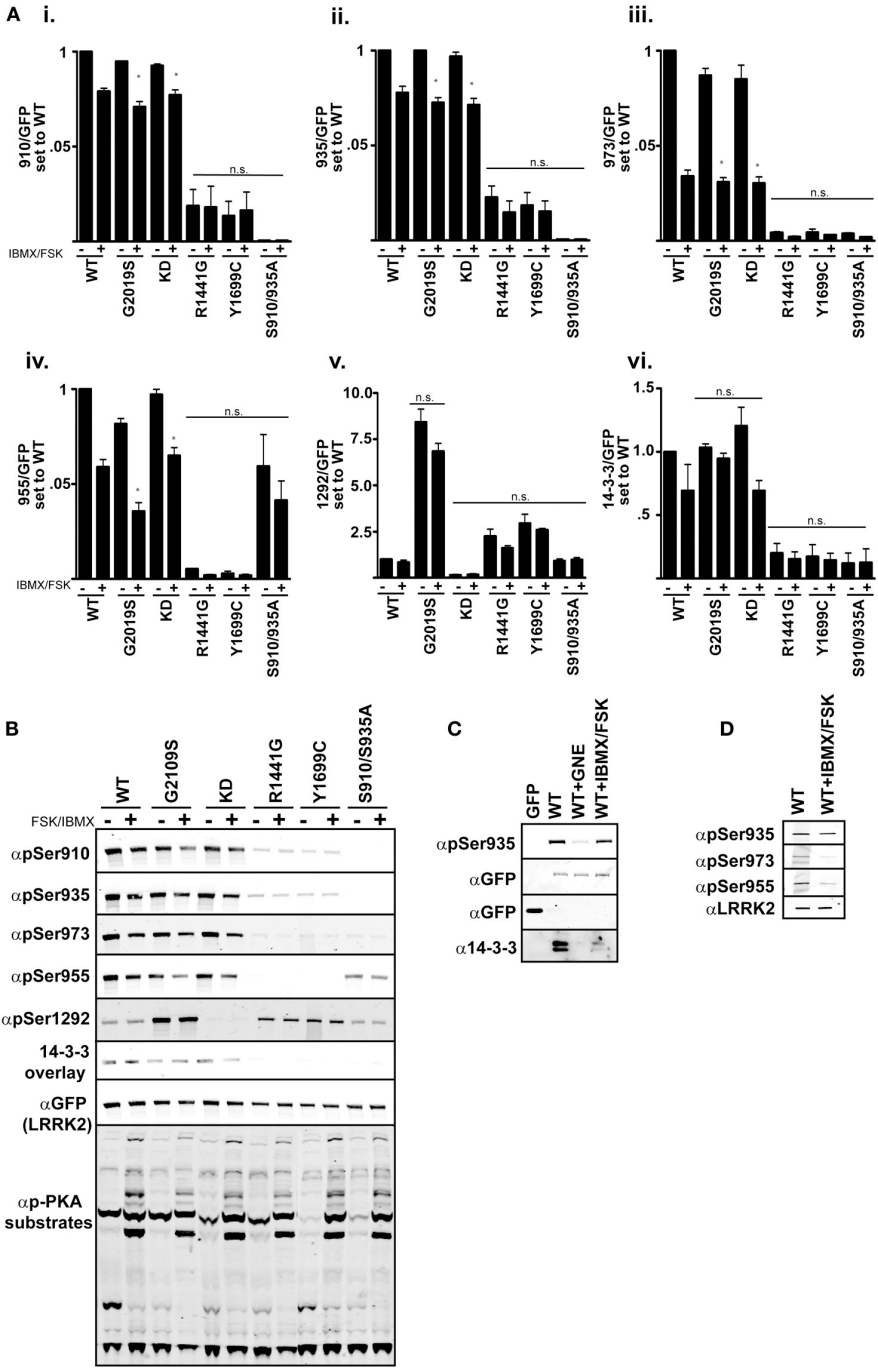
**cAMP stimulation does not increase 14-3-3 binding in HEK 293 cells. (Ai–vi)** Quantification of the phosphorylation levels at the cellular sites (935, 955, 973), autophosphorylation site 1292, and 14-3-3 binding in IBMX/FSK treatment relative to the DMSO treated control from **(B)**. **(B)** Representative blots of cellular site phosphorylation levels and autophosphorylation at Ser1292 in the indicated GFP-LRRK2 mutants after 30 min treatment with 50 μM FSK/100 μM IBMX. Stimulation of PKA activity is verified by immunoblotting cell lysates with phospho-PKA substrate antibody (Cell Signaling). Results are representative of 5 independent experiments for WT, Gly2019Ser, and KD mutants, and 2 independent experiments for the Arg1441Gly, Tyr1699Cys, and S910/935A mutants. The ratio of phosphorylation over total GFP signal was normalized to WT DMSO, error bars represent s.e.m. Statistical significance was assessed using the One-Way ANOVA test combined with Tukey’s correction. ^*^p ≤ 0.05. Quantification of WT samples treated with DMSO and IBMX/FSK are included in the graphs for reference, but were excluded in the statistical analysis. **(C)** LRRK2 interaction with 14-3-3 in cells is diminished by IBMX/FSK treatment. GFP or GFP-LRRK2 WT expressing HEK 293 T-REx cells were treated with GNE1023 for 90 min or 50 μM FSK/100 μM IBMX for 30 min, or in combination, then subjected to GFP Trap immunoprecipitation. Immunoprecipitates were immunoblotted for 14-3-3 co-immunoprecipitation. **(D)** Lung epithelial alveolar A549 cells were treated with 50 μM FSK/100 μM IBMX for 30 min and endogenous LRRK2 was immunoprecipitated and analyzed by immunoblot with anti-LRRK2 (N241), anti-phospho-Ser935, anti-phospho-955, anti-phospho-973 antibodies.

One model of LRRK2 regulation places the kinase domain downstream of the GTPase domain. In support of this, mutational analysis of the GTPase domain at Arg1441 appears to prolong the active state of LRRK2 by increasing its affinity for GTP and decreasing GTPase activity (Liao et al., 2014). Tyr1699Cys also decreases GTPase activity which could also lead to increased kinase activity (Daniels et al., 2011). We demonstrate in Figures 1–3, that these Roc-COR mutations increase kinase activity while strongly promoting dephosphorylation of the cellular sites. An alternate defect in LRRK2 regulation by Arg1441Gly was recently reported in Muda et al. (2014), where Ser1444 has been proposed as a new site of PKA phosphorylation that is required for 14-3-3 binding. *In vitro* Ser1444 phosphorylation and 14-3-3 binding are disrupted in the Arg1441Gly mutant, resulting in increased kinase activity in this model. LRRK2 was also found to intersect with PKA function by downregulating PKA activity in striatal projection neurons, where loss of LRRK2 or expression of a Arg1441Cys mutation leads to increased GluR1 and cofillin phosphorylation (Parisiadou et al., 2014). We therefore asked if activation of PKA, through modulating cAMP levels, changes LRRK2 phosphorylation at the cellular sites or activity in cells assayed through Ser1292. If PKA downregulates LRRK2 kinase activity and directly phosphorylates the cellular sites, then increasing PKA activity in cells would lead to decreased autophosphorylation of Ser1292 and increased phosphorylation at the cellular sites. We expressed GFP-LRRK2 WT, KD, Gly2019Ser, Arg1441Gly, Tyr1699Cys, Ser910/935Ala in HEK293 T-REx cells for 1 day, followed by 18 h of serum starvation then stimulation with Forskolin and IBMX for 30 min followed by immunoblot analysis Figure 4. These compounds increase cyclic AMP levels and block phosphodiesterase activity yielding increased PKA activity respectively, as indicated by increased PKA substrate antibody signal on whole cell lysates, Figure 4 quantitation in Figure 4A and immunoblot in Figure 4B. We found that all the cellular phosphorylation sites were significantly diminished by approximately 20% in a PKA activated environment and pSer1292 was not significantly changed. These data are in agreement with a recent report by Hermanson et al. (2012), which analyzed pSer935 levels following treatments with various LRRK2 inhibitors. In that report, PKA inhibitor H89 was not effective or had high cellular IC_50_ values compared other LRRK2 inhibitors in unstimulated HEK293 and U20S and SHSY5Y cells.

According to the model presented in Muda et al. (2014), PKA stimulated 14-3-3 binding negatively regulates LRRK2 activity and in the absence of this interaction activity would be increased. Thus, when PKA is activated, LRRK2 activity would be repressed. With the ability to assess LRRK2 activity directly in cells, we next asked if LRRK2 mutated in the Roc [Arg1441Gly] and COR [Tyr1699Cys] mutations, which would be deficient in cellular site phosphorylation, could regain 14-3-3 binding after Forskolin/IBMX treatment. The Ser910/935Ala mutant was included to detect PKA induced 14-3-3 binding independent of these sites. We observed no significant change *in vivo* LRRK2 kinase activity as indicated by Ser1292 phosphorylation after PKA stimulation, Figure 4B. This is consistent with previous reports that demonstrate the 14-3-3 interaction with LRRK2 is abolished by Ala substitution of Serines 910 and 935 in unstimulated conditions (Nichols et al., 2010). FSK/IBMX treatment also decreased the amount of LRRK2-14-3-3 complex in cells as demonstrated by co-immunoprecipitation, Figure 4C.

We wanted to confirm the effects of IBMX/FSK treatment in an additional cell line. LRRK2 knockout rodents develop abnormalities of the kidney and lung, showing accumulation of membranous materials and disruption of autophagy (Herzig et al., 2011; Baptista et al., 2013). A549 lung alveolar epithelial cells express significant amounts of endogenous LRRK2 (Lobbestael et al., 2013), and we used this cell system as a pertinent model of endogenous LRRK2 regulation. LRRK2 was immunoprecipitated from A549 cells after stimulation with IBMX/FSK, Figure 4D. Similar to the GFP expression system, we also observed a decrease in LRRK2 phosphorylation at the cellular sites. We were not able to detect a distinct pSer1292 on the endogenous LRRK2. Based on these results, the activation of LRRK2 by PKA may be specific to COS cells, which were used in Muda et al. Our experiments didn’t demonstrate a role for PKA in LRRK2 phosphorylation or autophosphorylation. In fact cAMP stimulation downregulated LRRK2 phosphorylation and suggested that a phosphatase may be activated in HEK293 and A549 cells.

#### Chemical analysis of ser910/935/955/973 and ser1292 phosphatases

**Figure 5:**
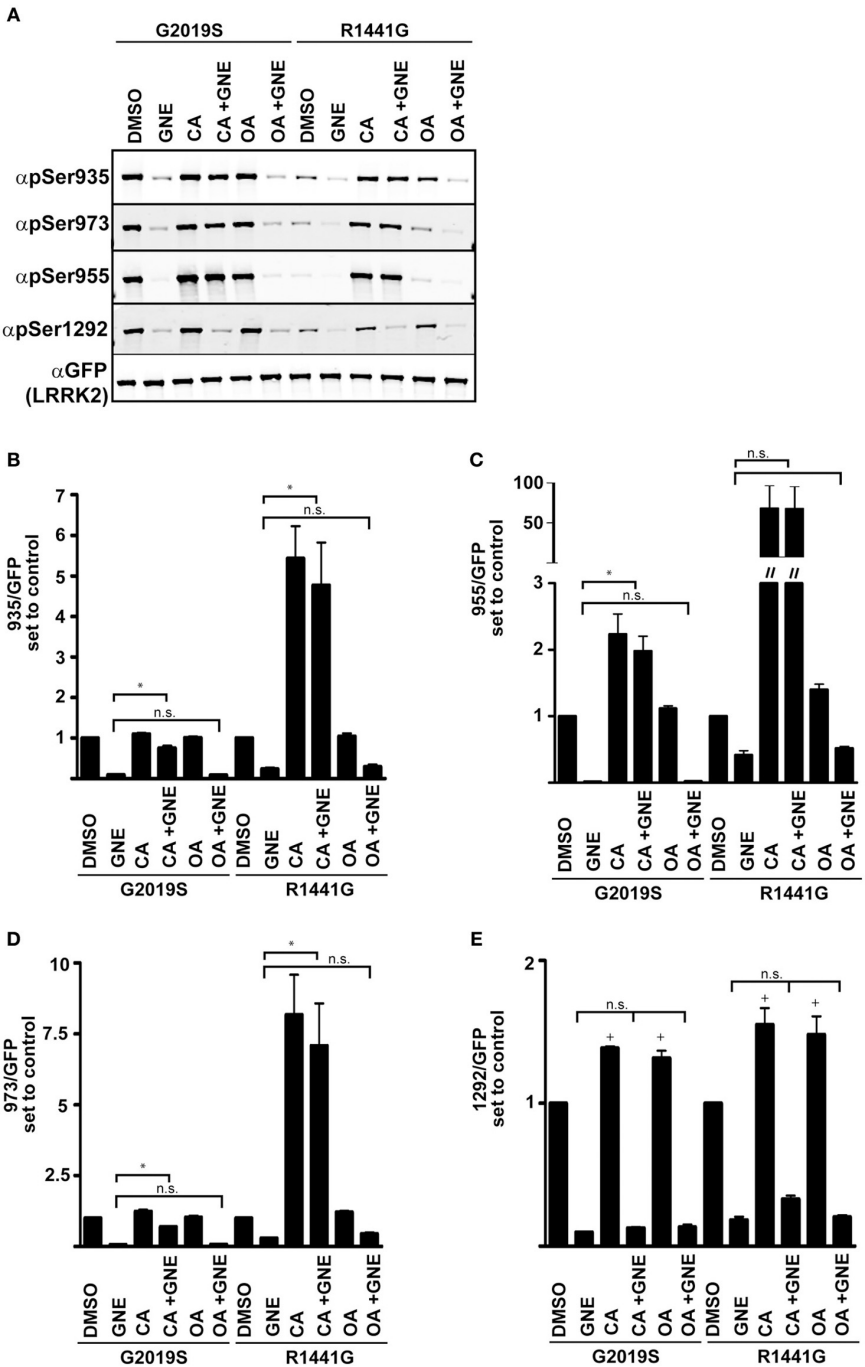
**Serine 1292 is regulated by Calyculin A and Okadaic acid sensitive phosphatases**. Stable-inducible HEK 293 T-REx cell lines overexpressing Gly2019Ser or Arg1441Gly LRRK2 mutation were treated with Okadaic acid or Calyculin A alone, or combined with GNE1023. **(A)** Representative blots of phosphorylation levels in Gly2019Ser and Arg1441Gly LRRK2 mutation line. **(B–E)** Quantification of anti-phospho-Ser935, anti-phospho-955, anti-phospho-973, and anti-phospho-1292 phosphorylation levels relative to the DMSO treated control of each mutant. The immunoblots shown are representative of three independent experiments. Statistical significance of Ser1292 stimulation with CA and OA was assessed using a one sample *t*-test set to the hypothetical value of 1, ^+^*p* ≤ 0.05. An ordinary One-Way ANOVA test combined with Dunnett’s correction for multiple testing was used to assess the ability of CA and OA to overcome GNE inhibition ^*^p ≤ 0.05 on connecting bars, n.s., not significant. DMSO control treated samples were not included in the ANOVA test and treatments were compared to GNE (alone) treated samples.

Phosphorylation of LRRK2 at the cellular sites and Ser1292 are efficient, although distinct indicators of LRRK2 kinase activity. The use of cellular phosphorylation sites as readouts of kinase activity in the Arg1441Gly, Arg1441Cys, Arg1441His, or Tyr1699Cys mutants is complicated by the increased interaction with phosphatase PP1, which leads to dephosphorylation of the cellular sites and reduced 14-3-3 interaction similar to treatment with LRRK2 inhibitors. Blocking PP1 activity with Calyculin A or expression of inhibitor proteins suppressed the induced dephosphorylation and inclusion body formation from kinase inhibition or PD mutations (Lobbestael et al., 2013). We assayed the sensitivity of Ser1292 to Calyculin A (CA) and Okadaic acid (OA), inhibitors of PP1 and PP2 respectively, in the context of LRRK2 inhibition and Arg1441Gly mutation, Figure 5. We expressed GFP-LRRK2 Gly2019Ser and Arg1441Gly in HEK293 T-REx cells in a doxycycline inducible manner for 1 day and treated cells with OA, CA, with and without GNE1023. We found that unlike Ser910/935/955/973, Ser1292 phosphorylation is sensitive to both CA and OA. Similar to previous reports, cellular site phosphorylation is rescued after inhibition of CA sensitive phosphatases, but not OA ones. Ser1292 phosphorylation is enhanced by both CA and OA treatment, indicating that LRRK2 autophosphorylation is regulated by both PP1 and PP2 type phosphatases. Unlike the cellular phosphorylation sites, Ser1292 phosphorylation is not restored by phosphatase inhibition after LRRK2 inhibition, confirming that Ser1292 is an autophosphorylation site and the cellular sites are regulated by upstream kinases.

## Discussion

Understanding how PD mutations in LRRK2 affect its biochemical properties and functional activities is essential to elucidation of how LRRK2 dysfunction causes neurodegenerative disease. The neurotoxicity of LRRK2 is connected with its kinase activity and mutants that increase kinase activity may be more pathogenic in patients. The GTPase and COR domains feed into the kinase domain and participate in LRRK2 regulation via intramolecular and intermolecular mechanisms (Biosa et al., 2013). By using mutations across the enzyme, we derive a better understanding of regulation of LRRK2 by itself and upstream enzymes. In this study, we used *in vitro* assays and expression systems to characterize the interplay of PD mutations and Ser910/935/955/973 phosphorylation and LRRK2 kinase activity that can be neurotoxic.

To gain insight into the effects of mutations in the Roc-COR and kinase domains on LRRK2 catalytic activity and regulation, we compared the detection of autophosphorylation sites in LRRK2 that fall between the amino terminal LRR and Roc domain [LRRK2-Ser1292], the Roc domain [LRRK2-Thr1491] and the carboxy terminal domain [LRRK2-Thr2483] to cellular site phosphorylation at Ser910, Ser935, Ser955, and Ser973. We employed a recombinant preparation of full length LRRK2 purified from mammalian cells, which provides an advantage over previous widely used preparations of recombinant forms lacking the amino terminus (LRRK2 aa970–2527) and purified from eukaryotic cells. This full length protein allows characterization of LRRK2 with all domains and their potential regulatory action in place. We compared phosphorylation of LRRKtide with autophosphorylation activity using pSer1292, pThr1491, pThr1503, and pThr2484 antibodies in wild-type, Arg1441Cys and Gly2019Ser mutants. LRRKtide and autophosphorylation activity saturate *in vitro* and are predictive for outcomes in cells. Out of these sites, only Ser1292 faithfully revealed active LRRK2 *in vitro* and in cells as an autophosphorylation site. Ile2012Thr suppressed kinase activity against Ser1292 and Thr1491 and also diminished the phosphorylation at the cellular sites.

Mutations Arg1441Cys, Arg1441Gly, Ala1442Pro, and Tyr1699Cys in the Roc-COR domain could activate LRRK2 kinase through structural changes that limit GTPase activity, prolonging the active state, leading to intramolecular stimulation of kinase activity (Taymans et al., 2011; Tsika and Moore, 2013; Liao et al., 2014). Recombinant Arg1441Cys exhibited increased kinase activity against LRRKtide, 20% above wild-type levels. Arg1441Cys, Arg1441Gly, Ala1442Pro, and Tyr1699Cys were dephosphorylated at the cellular sites, but are hyperphosphorylated at Ser1292 in cells. We did observe a larger effect of Tyr1699Cys on Ser1292 than previously described in Sheng et al. Threonine 1491 was evaluated in the presence of ATP and we found that this site revealed significant increase in kinase activity from the Gly2019Ser mutation. Interestingly, *in vitro* autophosphorylation at Thr2483 also revealed significant increased kinase activity from Arg1441Gly, and Gly2019Ser. The Tyr1699Cys mutation suppressed autophosphorylation of Thr1491 similar to what was observed for Thr1357 in Kamikawaji et al. (2013). Interrogation of individual autophosphorylation sites reveals different levels of mutation induced kinase activation, indicating that several sites should be analyzed simultaneously because these differences could be missed or misinterpreted by analysis of a single autophosphorylation site or total phosphate incorporation.

Protective LRRK2 haplotypes have been reported for Asn551Lys-Arg1398His-Leu1423Lys (Ross et al., 2011) and Arg1398His (Heckman et al., 2014), which further supports the notion that the amino terminus and the Roc-COR domain could feed into the kinase domain activity. When the cellular phosphorylation sites are mutated to unphosphorylatable Ala residues, we observed that mutation of Ser910/935, Ser955, and Ser973 to Ala did not affect intrinsic kinase activity in cells or on isolated LRRK2. However, mutation of all of the cellular sites to Ala trended toward slight increases in autophosphorylation activity of LRRK2 at Ser1292, and 2483, which could reflect regulatory activities of the amino terminal domain to the kinase domain through structural changes in the enzyme or through changes in subcellular localization. Increases in LRRK2 kinase activity from Arg1441Cys, Arg1441Gly, Ala1442Pro, and Tyr1699Cys PD mutations is not linked to the induced dephosphorylation of the cellular sites because alanine substitutions at serines 910/935/955/973 do not also increase LRRK2 kinase activity.

The increased phosphorylation of Ser1292 compared to the decreased phosphorylation of the cellular sites in Arg1441Cys, Arg1441Gly, Ala1442Pro, and Tyr1699Cys mutants indicated that there may be different phosphatases acting on these sites in addition to increasing the kinase activity of LRRK2. To understand the class of phosphatase that act on Ser1292 compared to the cellular sites, we utilized Calyculin A and Okadaic acid, pharmacological inhibitors of PP1 and PP2, respectively. Ser1292 dephosphorylation is mediated by both CA and OA sensitive phosphatases, while the cellular sites are sensitive to only CA sensitive phosphatases. This supports the notion that indeed Ser1292 is dephosphorylated by a different phosphatase class than the cellular sites. Ser1292 is a bona fide autophosphorylation site *in vivo* because it does not become re-phosphorylated after inhibition of both LRRK2 kinase and the phosphatase. This side-by-side comparison also validates that Ser910/935/955/973 are not phosphorylated by LRRK2 like the direct autophosphorylation site Ser1292, Thr1491, and Thr2483. Though the Ser1292 site is detected in LRRK2 isolated from cells it is thought to be of low stoichiometry with the total amount of LRRK2. The fact that Thr1491 or Thr2483 were not detectable in cells may be due to low activity against these sites in cells or increased phosphatase activity against these sites in cells. Alanine substitution of Ser1292 causes a decrease in the cytoplasmic filaments and an increase in the cytoplasmic puncta caused by Arg1441Gly mutations (Sheng et al., 2012). The different phosphatases that target both Ser1292 and serines 910/935/955/973 are presumably more active on LRRK2 that has been inactivated through inhibition. In the future it would be important to elucidate the Ser1292 phosphatase and/or the phosphatase regulatory subunits that regulate the phosphatase activity on LRRK2.

Phosphorylation of Ser1292 is a biochemical readout of LRRK2 activity in cells and *in vitro*. LRRK2 inhibitor induced dephosphorylation of LRRK2 cellular sites Ser910/935/955/973 and Ser1292 are observed at similar IC50s, indicating that both are measures of activity. We were also able to detect increased Ser1292 activity in a Gly2019Ser heterozygous patient derived lymphoblastoid culture, and that Ser935/955/973 as well as Ser1292 were efficiently dephosphorylated in these cells upon GNE treatment, suggesting that Ser1292 can be employed as a measure of LRRK2 activity in patients. High levels of LRRK2 expression in the periphery and responsiveness of the cellular sites and Ser1292 to specific LRRK2 inhibitors provides a tractable patient sample source to ascertain and analyze LRRK2 modification in longitudinal studies or as a surrogate marker for targeting LRRK2 during inhibitor based trials.

We next explored the effects of modulating cAMP induced PKA activity on LRRK2 autocatalytic activity and cellular phosphorylation. We found that Forskolin/IBMX treatment resulted in a consistent diminution of both the cellular sites and Ser1292, and no change in 14-3-3 binding. Though contradictory to the report in Muda et al. (2014), these data are consistent with previous reports that loss of either Ser910 or Ser935 phosphorylation does not change kinase activity but does result is loss of 14-3-3 binding in cells and in overlay far-western assays. Furthermore, treatment of cells with H89, a PKA inhibitor does not reduce LRRK2 phosphorylation at Ser935 (Hermanson et al., 2012).

**Figure 6:**
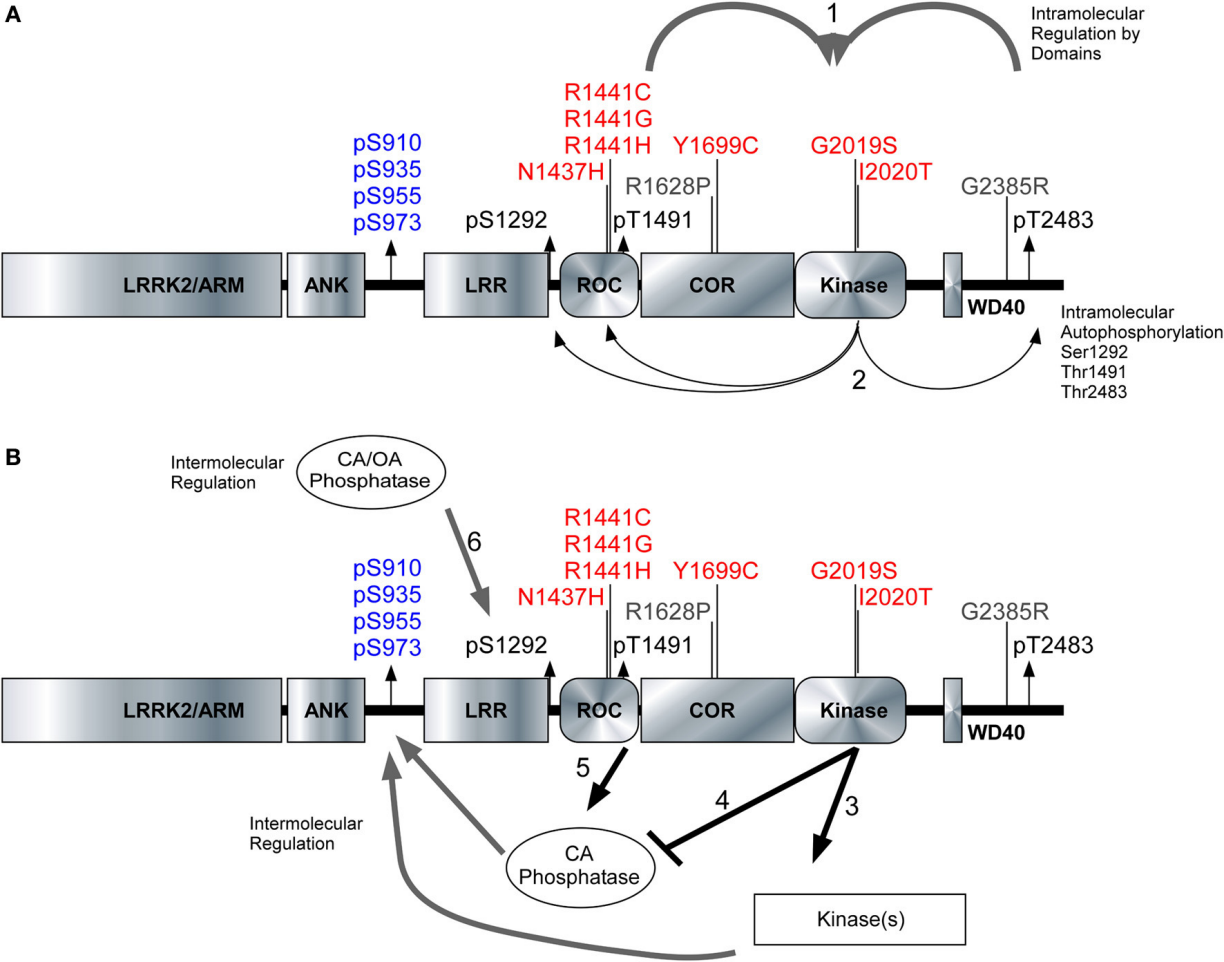
**Model of intramolecular and intermolecular LRRK2 regulation**. The LRRK2 structure with PD associated mutants highlighted in red (pathogenic) and risk factors in gray. Cellular phosphorylation sites are highlighted in blue and autophosphorylation sites are in black. **(A1)** The intramolecular regulation of the PD associated mutants in the Roc-COR domain and the carboxy terminus on LRRK2 kinase activity. **(A2)** Differential effects of the PD mutations can be detected through the autophosphorylation sites Ser1292, Thr1491, and Ser2483. **(B3,B4)** Kinases and phosphatases contribute to the intermolecular regulation of LRRK2 and their effects can be demonstrated through cellular phosphorylation sites and autophosphorylation sites. **(B5)** Roc-COR mutations enhance the dephosphorylation of LRRK2 at the cellular sites through increased interaction with phosphatases. Ser1292 is dephosphorylated by Okadaic acid and Calyculin A sensitive phosphatases, **(B6)** Mutations within the Roc-COR allow for increased interactions with phosphatases and this decreases the cellular phosphorylation levels.

We characterized three autoregulatory phosphosites [Ser1292, Thr1491, and Thr2483] alongside the cellular phosphorylation sites [Ser910/935/955/973] to reveal true differences in how pathogenic PD mutations affect the activity and phosphorylation status of LRRK2. The kinase domain of LRRK2 is peculiarly packed amongst several structural and enzymatic domains that influence the kinase activity of LRRK2. Figure 6A illustrates the domain structure of LRRK2, along with the position of PD mutations, autophosphorylation sites and the cellular phosphorylation sites. Roc-COR mutations can increase the kinase activity through intramolecular effects (Figure 6A1) as indicated by autophosphorylation sites Figure 6A2. Figure 6B depicts the proposed regulation of LRRK2 phosphorylation at the cellular phosphorylation sites and Ser1292 by upstream kinases (Figure 6B3) and phosphatases (Figure 6B4), which could be up and down-regulated by LRRK2, respectively. At the same time, Roc-COR mutations enhance the dephosphorylation of LRRK2 at the cellular sites through increased interaction with phosphatases, Figure 6B5. Ser1292 is dephosphorylated by Okadaic acid and Calyculin A sensitive phosphatases, Figure 6B6. LRRK2 autophosphorylates each site differently dependent on mutation and these autophosphorylation sites indicate LRRK2 activity different from the cellular sites. Further investigation of the phosphoregulation of LRRK2 will continue to provide novel insight into the normal activities of the enzyme as well as how PD mutations may disrupt enzyme function.

### Conflict of interest statement

R. Jeremy Nichols is a consultant for Thermo Fisher. Steve M. Riddle and Connie S. Lebakken are both employees of Thermo Fisher. Thermo Fisher had no role in the design or implementation of the study.
