# Supplementary material for: LRRK2 kinase activity and biology are not uniformly predicted by its autophosphorylation and cellular phosphorylation site status
Source: Front Mol Neurosci. 2014 Jun 24;7:54. doi: 10.3389/fnmol.2014.00054 (PMC4068021; doi:10.3389/fnmol.2014.00054)
Supplement: Supplementary file 1 [file DataSheet1.PDF]

Table 1. Characterized LRRK2 autophosphorylation sites

Autophosphorylation site	Greggio, 2008	Greggio, 2009	Kamikawaji, 2009	Glocker, 2010	Kamikawaji, 2013	Pungaliya, 2010	Webber, 2011	Li, 2010	Sheng, 2012	Comments	Antibody available
S4/S5				x							
T424				x							
T524				x							
T776				x							
T826				x							
T833				x							
T838				x							
T1024/S1025		x									
S1124				x							
S1292				x					x	Phosphorylated in cells	**Sheng et al. 2013**
T1343		x		x			x		x		
S1345		x		x		x					
T1348/T1349		x		x	x		x			Putative major phosphorylation sites	
T1357					x		x			Putative major phosphorylation site	**Kamikawaji, 2013**
T1368				x			x		x		
S1403/S1404			x						x		
T1410			x	x		x					**Abcam**
T1452				x							
T1491		x	x	x			x				**Abcam**
T1967/T1969			x							Ala substitution decreases kinase activit	**Kamikawaji, 2009**
T1503				x			x			Modification disrupts GTPase function	**Abcam**
T2031/S2032	x	x						x			
T2035	x							x		Putative T-loop phoshorylation sites	
T2483				x							**Abcam**

Supplemental Figure 1

A.

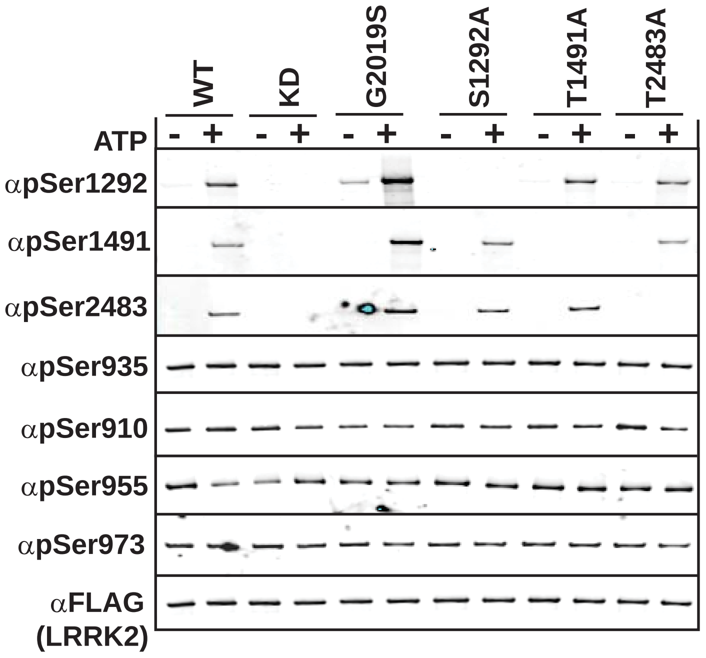

Supplemental Figure 1. **Validation of autophosphorylation site antibodies.** (A) HEK-293 cells transiently expressing the indicated FLAG-tagged forms of LRRK2 were subjected to immunoprecipitation kinase assays using anti FLAG M2 agarose, in the presense (+) or absence (-) of ATP. Reaction products were probed with anti-phospho-Thr1491 (αpThr1491), anti-phospho-Ser1292 (αpS1292), anti-phospho-Thr2483 (αpThr2483), anti-phospho-Ser935 (αpS935), anti-phospho-Ser910 (αpS910), anti-phospho-Ser955 (αpS955), anti-phospho-Ser973 (αp973). Blots were probed with anti-FLAG (αFLAG) for total protein control.

Supplemental Figure 2

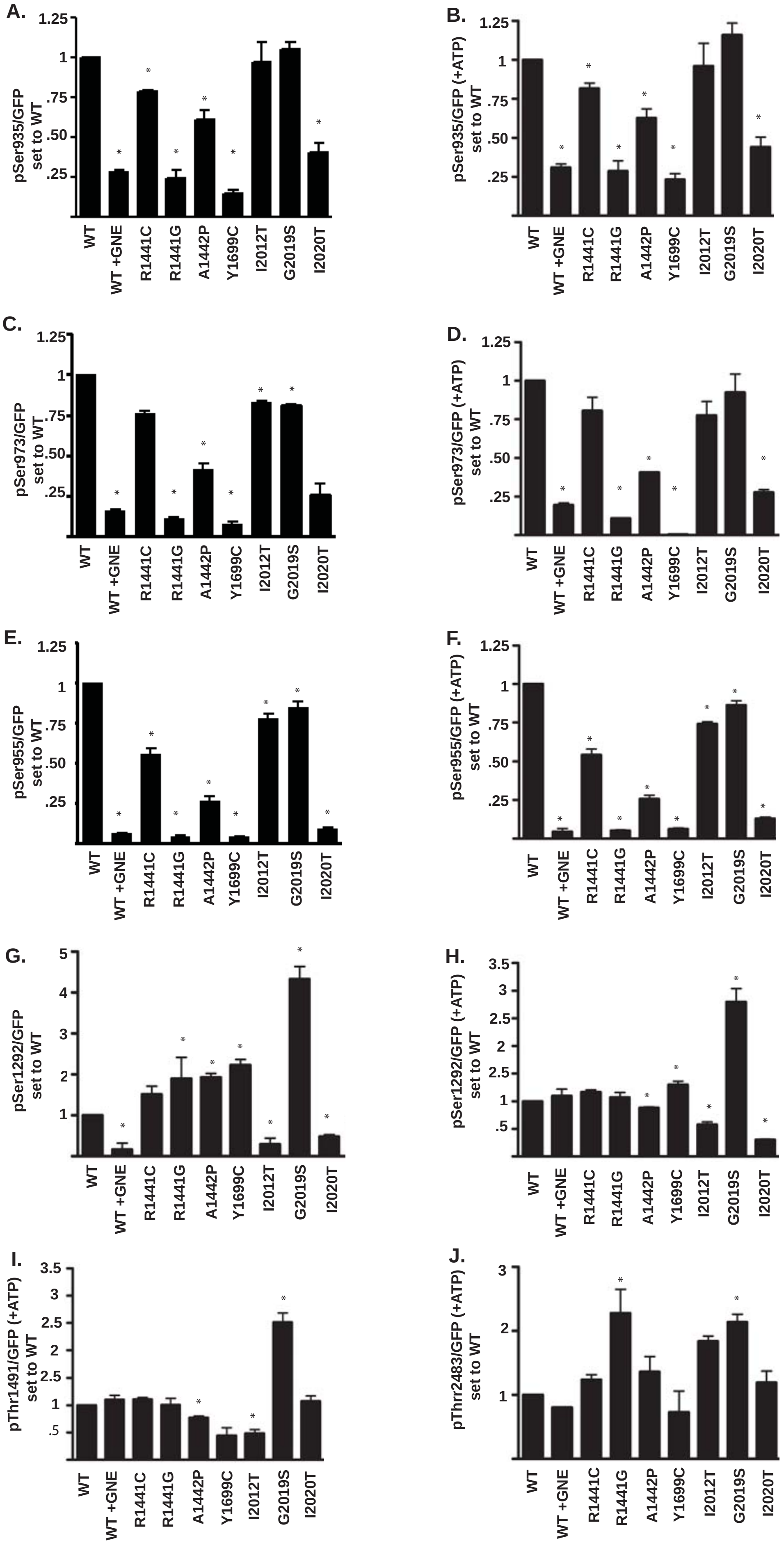

Supplemental Figure 2

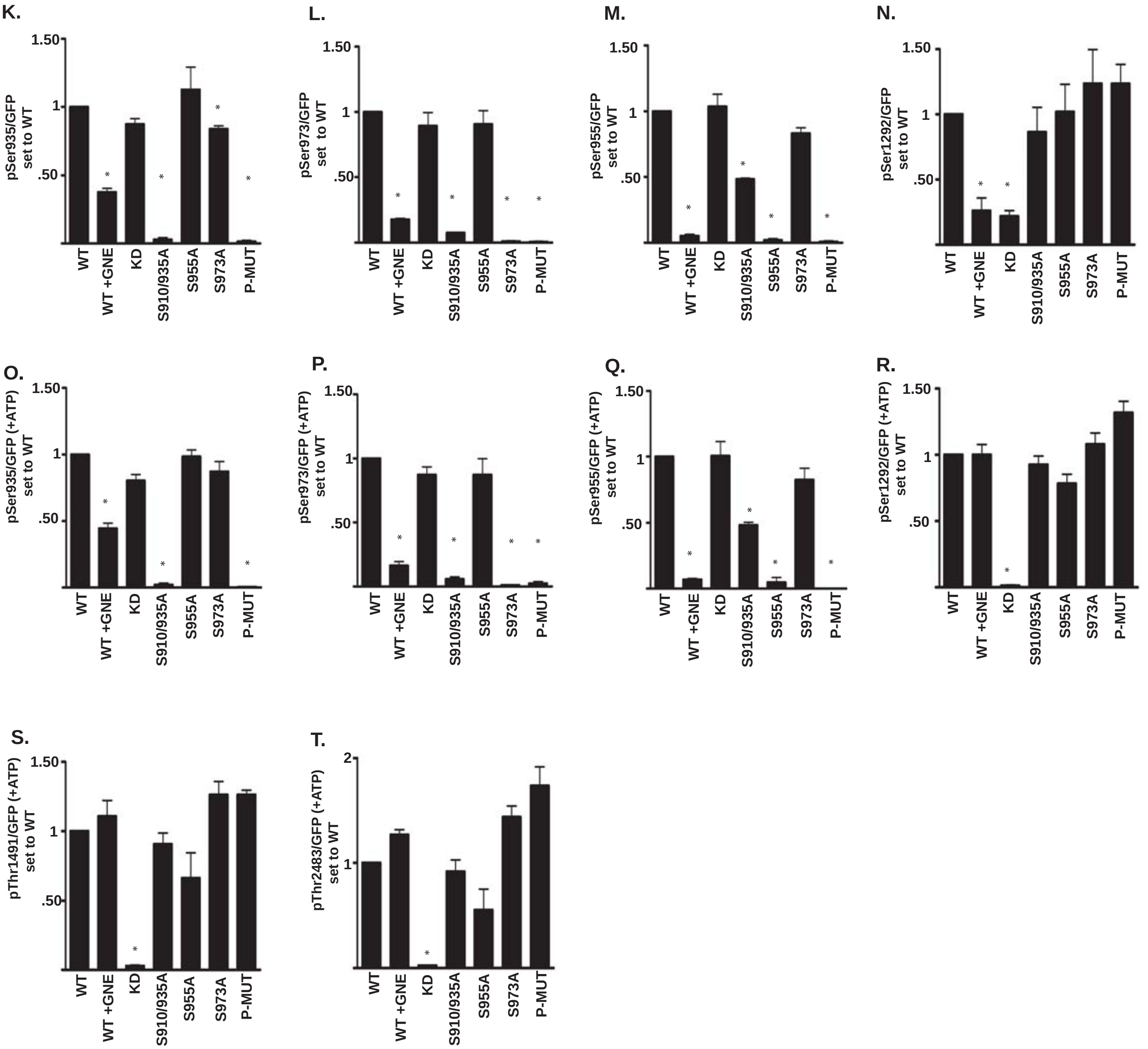

Supplemental Figure 2. **Quantification of phosphorylation signal at the cellular phosphorylation and autophosphorylation sites in the presence and absence of ATP**. (A-F) Quantification of the cellular phosphorylation sites in WT and the PD associated mutants. Basal activity (-ATP) for 935, 973, and 955 is shown in graphs A, C, E respectively and +ATP activity is shown in graphs B,D,F. (G-J) Quantification of autophosphorylation sites in WT and PD associated mutants. Basal activity (-ATP) is only shown for pSer1292 (G). Activity assessed +ATP is shown for 1292, 1491, and 2483 in graphs H, I, J respectively. Quantification of the cellular phosphorylation sites in phosphosite mutants. Basal activity (K-M) and +ATP activity (O-Q) is shown for 935, 973, 955 respectively. Quantification of autophosphorylation site 1292 levels (basal and +ATP activity) are shown in N and R, respectively and +ATP activity of 1491 and 2483 are shown in S and T respectively. For quantification, the ratio of phosphorylation signal over total GFP signal was normalized to WT or WT +ATP control. The immunoblots and graphs shown are representative of three independent experiments for the all lines, except the analysis of pThr2483 is of two independent experiments for I2020T, G2019S, I2012T, and G2385R. Statistical significance was assessed using the one sample *t* test set to the hypothetical value of 1. Error bars represent s.e.m, * p<.05.

Supplemental Figure 3

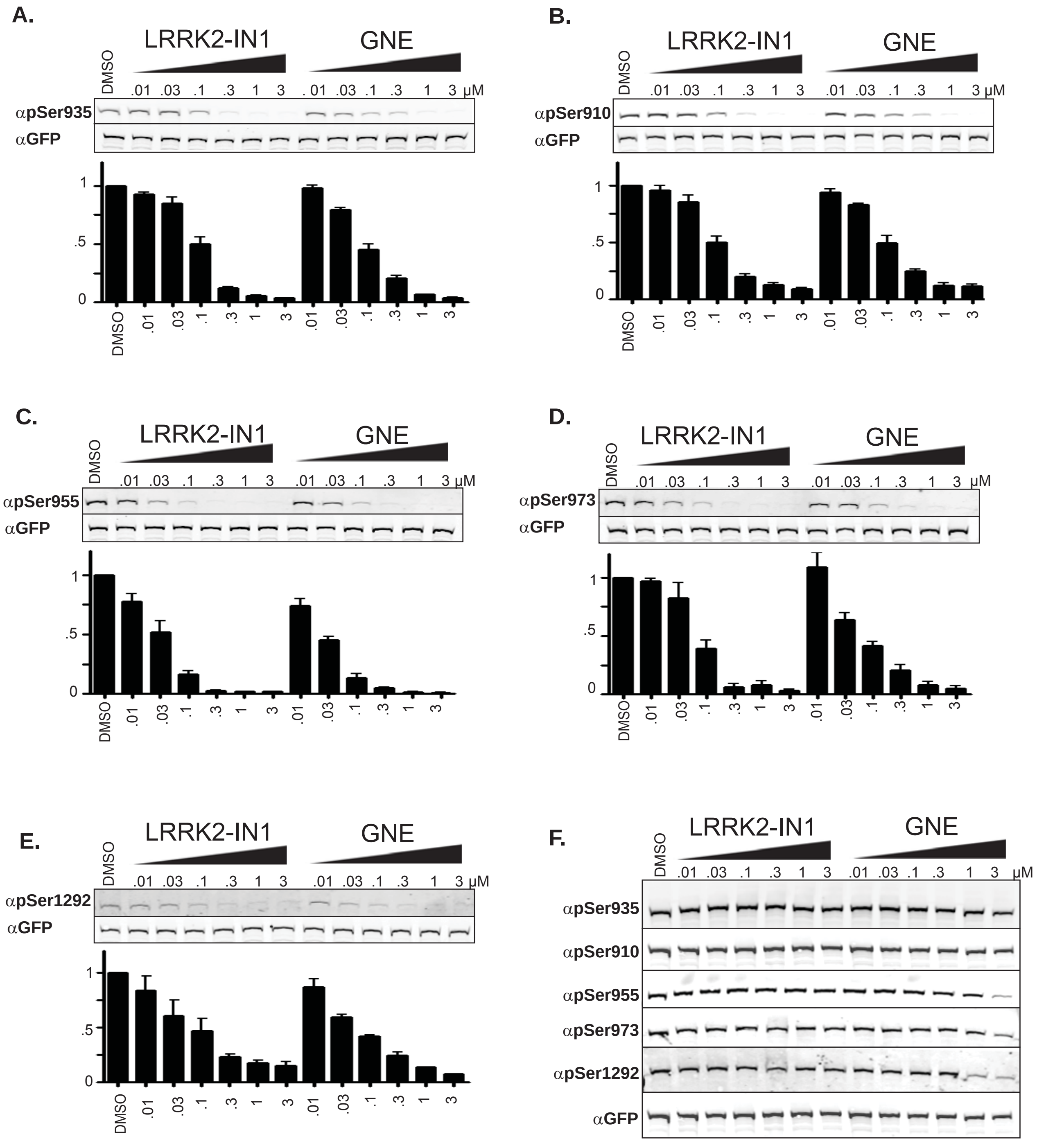

Supplemental Figure 3. Comparative Analysis of inhibitor induced dephosphorylation for Ser1292 and cellular phosphorylation sites after kinase inhibition. HEK293 T-REx cells expressing GFP-LRRK2 [G2019S] were treated with the indicated concentrations of LRRK2-IN1 or GNE1023 for 90 minutes. Cell lysates were immunoblotted for detection of cellular phosphorylation sites (A) anti-phospho-Ser935 (αpS935), (B) anti-phospho-Ser910 (αpS910), (C) anti-phospho-Ser955 (αpS955), (D) anti-phospho-Ser973 (αp973), (E) anti-phospho-Ser1292 (αpS1292). The immunoblots shown are representative of three independent experiments. The results are presented as percentage of kinase activity relative to the DMSO treated control. IC50 values were derived using curve fitting in GraphPad Prism. (F) HEK293 T-REx cells expressing the inhibitor resistant mutant GFP-LRRK2 [A2016T/G2019S] were treated and analyzed similar to (A-E).
